# Eosinophilic Granulomatosis With Polyangiitis With Thrombotic Microangiopathy: Is Simultaneous Systemic Lupus Erythematosus Associated With Clinical Manifestations? A Case Report and Review of the Literature

**DOI:** 10.1097/MD.0000000000001943

**Published:** 2015-11-13

**Authors:** Shoichi Fukui, Naoki Iwamoto, Sosuke Tsuji, Masataka Umeda, Ayako Nishino, Yoshikazu Nakashima, Takahisa Suzuki, Yoshiro Horai, Tomohiro Koga, Shin-ya Kawashiri, Kunihiro Ichinose, Yasuko Hirai, Mami Tamai, Hideki Nakamura, Tomoki Origuchi, Naóe Kinoshita, Atsushi Kawakami

**Affiliations:** From the Department of Immunology and Rheumatology (SF, NI, ST, MU, AN, YN, TS, YH, TK, S-YK, KI, YH, MT, HN, TO, AK); Department of Public Health (S-YK); Department of Rehabilitation Sciences, Nagasaki University Graduate School of Biomedical Sciences (TO); and Department of Pathology, Nagasaki University Hospital, Nagasaki, Japan (NK).

## Abstract

Eosinophilic granulomatosis with polyangiitis (EGPA) is one of the antineutrophil cytoplasmic antibody (ANCA)-associated vasculitis, which is characterized by vasculitis of the small to medium-sized vessels. On the contrary, thrombotic microangiopathy (TMA) is a life-threatening condition which can cause ischemic organ injury. Although several case reports have described patients with TMA associated with ANCA-associated vasculitis except for EGPA, there are no previous case reports of EGPA associated with TMA.

A 71-year-old Japanese man was diagnosed with EGPA based on his asthma, eosinophilia, lung opacity, refractory sinusitis, and positive myeloperoxidase-ANCA. He was also diagnosed with TMA based on peripheral schizocytes and hemolytic anemia. We performed plasmapheresis and started high-dose corticosteroid therapy; thereafter, he improved promptly. His case also fulfilled the classification criteria of systemic lupus erythematosus (SLE) based on the pleural effusion, renal disorder, anemia, thrombocytopenia, positive antidouble-stranded DNA antibody, and low complement. Elements of SLE were thought to affect his clinical course.

We reviewed 11 patients with EGPA or hypereosinophilic syndrome (HES) associated with SLE, including our case. Patients with EGPA or HES associated with SLE had more heart complications than patients with simple EGPA or simple HES did. Patients with EGPA or HES associated with SLE had more pleural effusion than patients with simple SLE did.

Clinical manifestations of eosinophilia with SLE or SLE with eosinophilia may differ from simple SLE or simple eosinophilia.

## INTRODUCTION

Antineutrophil cytoplasmic antibody (ANCA)-associated vasculitis (AAV) is characterized by vasculitis of the small to medium-sized vessels; however, its etiology remains unknown. There are three types of AAV: eosinophilic granulomatosis with polyangiitis (EGPA), granulomatosis with polyangiitis (GPA), and microscopic polyangiitis (MPA). On the contrary, thrombotic microangiopathy (TMA) is characterized by thrombocytopenia and microangiopathic hemolytic anemia, which can cause ischemic organ injury of brain, kidneys, heart, pancreas, liver, and lungs. TMA is a very severe disease and needs therapeutic options such as plasmapheresis.

Several case reports have described patients with MPA and GPA associated with TMA. Regarding only MPA and GPA, 14% of patients are reported to have TMA.^1^ However, EGPA associated with TMA have not ever been reported.

We present a case of EGPA with TMA, which was thought to be associated with elements of SLE including hypocomplementemia.

## CASE PRESENTATION

Two months before his admission to our hospital, the thrombocytopenia (80,000/μL) of a 71-year-old Japanese man was identified by a regular medical examination. Two weeks before his admission to our hospital, his platelet count declined to 20,000/μL, and petechia and palpable purpura emerged on both legs. On admission to our hospital, his dyspnea was observed all day. Eleven years earlier, he was diagnosed with bronchial asthma and was prescribed inhaled glucocorticoids. He had also undergone an operation for bilateral sinusitis of maxillary sinuses 2 years before this admission. He was a psychiatrist. He did not have any family history of rheumatic diseases.

**FIGURE 1:**
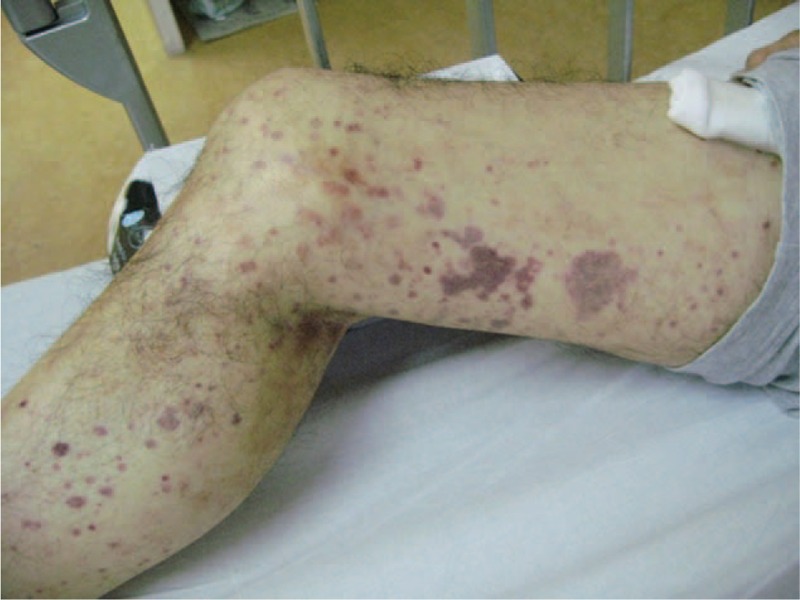
Petechia and palpable purpura on both legs.

The physical examination results at the present admission were as follows: body temperature 37.8°C; blood pressure 122/82 mm Hg; pulse rate 100/min; and respiratory rate 30/min. Auscultation of the chest revealed wheezing over the entire lung field. There was pitting edema in the both lower legs. Petechia and palpable purpura on both legs were seen (Fig. 1).

The laboratory data were as follows: white blood cell count 16,900/μL (neutrophils 28%, lymphocytes 10%, monocytes 2%, eosinophils 59%, and basophils 1%); hemoglobin 7.5 g/dL; platelets 16,000/μL; C-reactive protein 2.38 mg/dL (normal values <0.3 mg/dL); serum creatinine (Cr) 0.87 mg/dL (normal values <1.0 mg/dL); rheumatoid factor 5 IU/mL (normal values <15 IU/mL); antidouble-stranded DNA antibody (anti-dsDNA antibody) (enzyme-linked immunosorbent assay) 27.4 IU/mL (normal values <12 IU/mL); and a high myeloperoxidase-antineutrophil cytoplasmic autoantibody (MPO-ANCA) level of 10.2 enzyme-linked immunosorbent assay units (EU)/mL (normal values <3.5 EU/mL); immunoglobulin E 3017 IU/mL (normal values <173 IU/mL).

Proteinase 3-ANCA was negative. Antinuclear antibody (ANA) (immunofluorescence assay on Hep-2 cell) was not detected. He also had hypocomplementemia (complement activity [CH50]: 6.1 CH50/mL [normal range: 25.0–48.0 CH50/mL], complement component 3 (C3): 40.0 mg/dL [normal range: 86–160 mg/dL], complement component 4: 6.3 mg/dL [normal range: 17–45 mg/dL]) and low haptoglobin (<5.0 mg/dL [normal range: 83–209 mg/dL]). A peripheral blood smear showed schizocytes. A disintegrin-like and metalloproteinase with thrombospondin type 1 motifs 13 (ADAMTS13) activity was 49.8% (normal range: 70.0%–120.0%) and ADAMTS13 inhibitor was negative. Urinalysis revealed microscopic hematuria (11–20 red blood cells/high-power field) and proteinuria (0.74 g/g Cr), but no type of casts. Head computed tomography (CT) showed bilateral sinusitis of maxillary sinuses.

**FIGURE 2:**
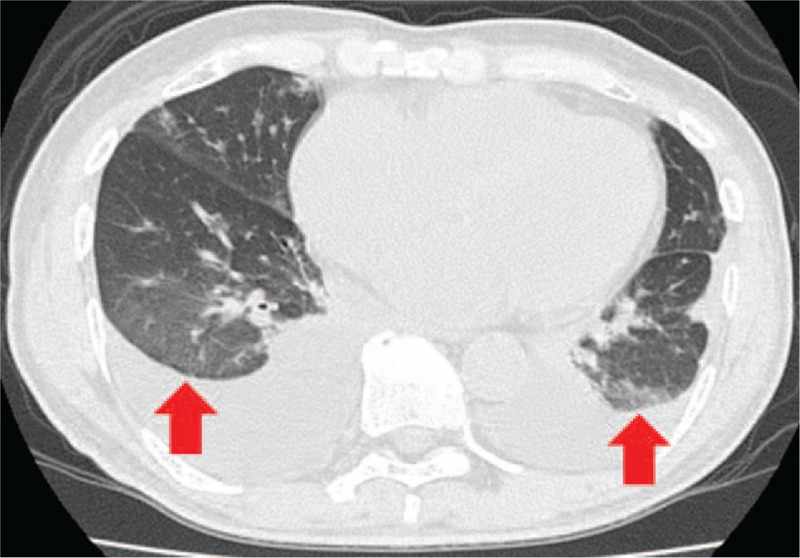
Chest CT showed peripheral ground glass opacity (arrow), dilated heart, and large amount of pleural effusion. CT = computed tomography.

**FIGURE 3:**
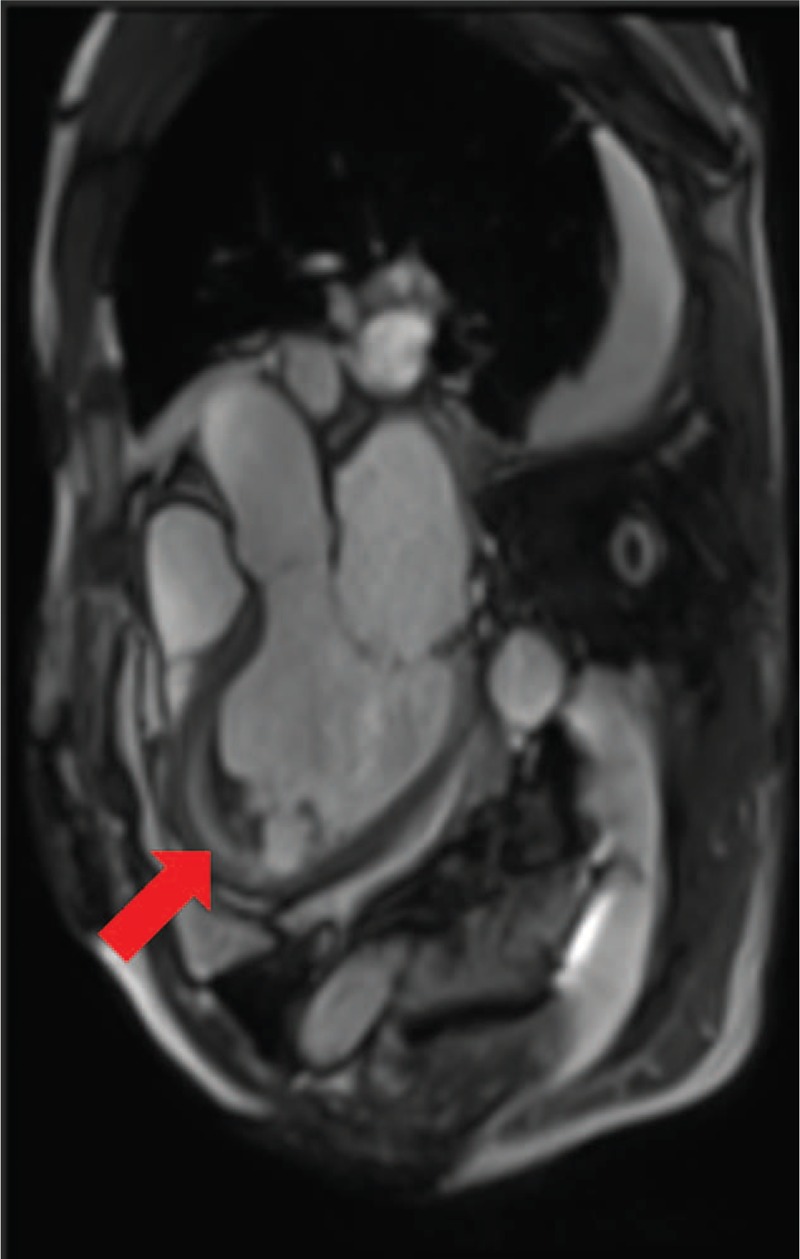
MR image of heart showed intraventricular thrombus (arrow) and thin endocardium with high-intensity signal. MR = magnetic resonance.

A chest CT showed peripheral ground glass opacity, dilated heart, and large amount of pleural effusion (Fig. 2). A magnetic resonance image (MRI) of the patient's heart showed intraventricular thrombus and thin endocardium with high-intensity signal (Fig. 3). A bone marrow specimen showed prominent eosinophilic bone marrow. FIP1L1-PDGFRA fusion gene and t(5;12)(q31–q33;p12), which is often observed in myeloproliferative neoplasms with prominent eosinophilia, were both negative.

The patient's poor condition and platelet decline precluded renal biopsy. Because he had asthma, eosinophilia >10%, pulmonary infiltrates (non-fixed), and paranasal sinus abnormality, a diagnosis of EGPA was made, based on the Chapel Hill Consensus Conference criteria,^2^ the European Medicines Agency algorithm,^3^ and the American College of Rheumatology (ACR) 1990 criteria for the classification of Churg–Strauss syndrome.^4^ In addition, because the patient had schizocytes, hemolytic anemia, thrombocytopenia, and renal insufficiency, we also diagnosed TMA. Given the pleural effusion, renal disorder, anemia, thrombocytopenia, positive anti-dsDNA antibody, and low complement, this patient also fulfilled the 1997 American College of Rheumatology classification criteria^5^ and the 2012 Systemic Lupus International Collaborating Clinics classification criteria^6^ for systemic lupus erythematosus (SLE).

Because his dyspnea worsened progressively day by day and we suspected that he could develop cardiac failure associated with EGPA, we started pulsed methylprednisolone (P-MPSL; 1000 mg/d for 3 consecutive days) and plasmapheresis. Soon after these treatments, the eosinophils disappeared and the patient's dyspnea was improved. Regarding the TMA, the schizocytes disappeared and hemolytic anemia improved. We finished the plasmapheresis after only 2 consecutive days.

**FIGURE 4:**
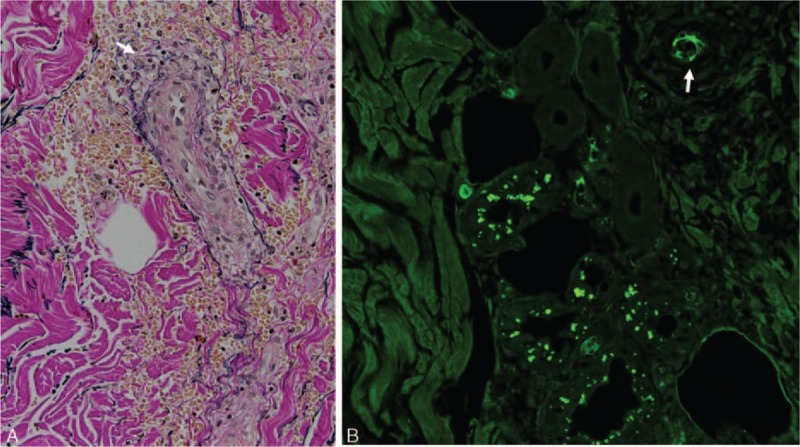
A, Fragmentation of the lamina elastica interna in the skin biopsy specimen (white arrow). B, Immunohistochemical staining of complement factor C3 showed a deposition of C3 in the vessel wall (white arrow).

The analysis of a skin biopsy specimen 3 days after the P-MPSL was started revealed fragmentation of the lamina elastica interna (Fig. 4A). Immunohistochemical staining of complement factor C3 showed a deposition of C3 in the vessel wall (Fig. 4B).

Following the P-MPSL, we started 55 mg of prednisolone per day, and the patient's MPO-ANCA titer and anti-dsDNA antibody declined gradually. The endocardium with high-intensity signal on MRI was diminished 3 weeks after the start of the P-MPSL. The patient's hypocomplementemia also improved. Eosinophils remained negative.

## DISCUSSION

We have described the case of a patient with EGPA associated with TMA. To our knowledge, this is the first case report of EGPA associated with TMA. Chen et al^1^ reported that of 220 patients with AAV excluding EGPA, 30 patients (14%) had concomitant renal TMA by pathologic evaluation, and AAV with TMA was not rare. Using PubMed, Manenti et al^7^ reviewed 29 cases with AAV with TMA, and they argued that mutations or risk haplotypes in genes encoding alternative complement regulatory proteins associated with TMA. Thurman et al^8^ reported that patients with acute-onset diarrhea-associated hemolytic uremic syndrome manifested activation of the alternative pathway of complement that is temporally related to the onset of disease.

In our patient, a skin biopsy revealed C3 deposition on the vessel wall. Magro et al^9^ reported that a skin biopsy is of value in establishing a diagnosis of TMA based on the microvascular deposition of complement in the dermis and subcutaneous fat. Because hypocomplementemia also existed in our patient, an alternative pathway of complement may have been associated with his TMA. Nevertheless, this skin biopsy finding seems incompatible to AAV because AAV is defined as pauci-immune vasculitis. However, Brons et al^10^ reported that the skin lesions of patients with GPA had immune deposits.

Moreover, Chhabra et al^11^ revealed that the observation of immune deposits in cutaneous lesions of GPA is useful as a predictor of an active disease. Although to the best of our knowledge, there are no case reports of EGPA with C3 or immune deposits in skin biopsy, an alternative pathway may have been associated with the occurrence of TMA in our patient.

Our patient also fulfilled the criteria for SLE, which is also associated with TMA.^12^ SLE with negative ANA and positive anti-dsDNA antibody is thought to be uncommon in general. However, Almogren et al^13^ reported that of 158 patients who were simultaneously tested ANA (immunofluorescence assay on Hep-2 cell) and anti-dsDNA antibody (immuno-enzymatic assay), 24 (15.1%) patients had statuses which were negative ANA and positive anti-dsDNA antibody. In addition, Craig and Ledue^14^ reported that 2 patients had positive anti-dsDNA antibody (Crithidia luciliae immunofluorescence test) among 72 patients with SLE that had negative ANA (immunofluorescence assay on Hep-2 cell) statuses. International recommendations for the assessment of autoantibodies to cellular antigens referred to as antinuclear antibodies^15^ says: “Theoretically, if ANA-indirect immunofluorescence assay is negative, one should not proceed to defining anti-dsDNA antibodies, although if clinical suspicion of SLE is substantial anti-dsDNA antibodies assessment may be requested by the clinician.” On the basis of these facts, we think that it does not matter to diagnose our case with SLE.

Regarding complement, Song et al^16^ reported that complement overactivation via both classical and alternative pathways might play an important role in the pathogenesis of renal TMA in lupus nephritis. Eosinophils are rarely seen in SLE. We searched for case reports of EGPA or hypereosinophilic syndrome (HES) associated with SLE by conducting a PubMed search. Table 1  shows the results: 11 patients with EGPA or HES associated with SLE, including our case. We could find only 2 cases of SLE with EGPA, including our case. D’Cruz et al^17^ reported a patient who, after 26 years of SLE, developed late-onset asthma and eosinophilia, which progressed to meet the ACR 1990 criteria for the classification of Churg–Strauss syndrome. However, except for fulfilling the Churg–Strauss syndrome criteria, D’Cruz et al's patient did not have features in common with those of our patient.

**TABLE 1:**
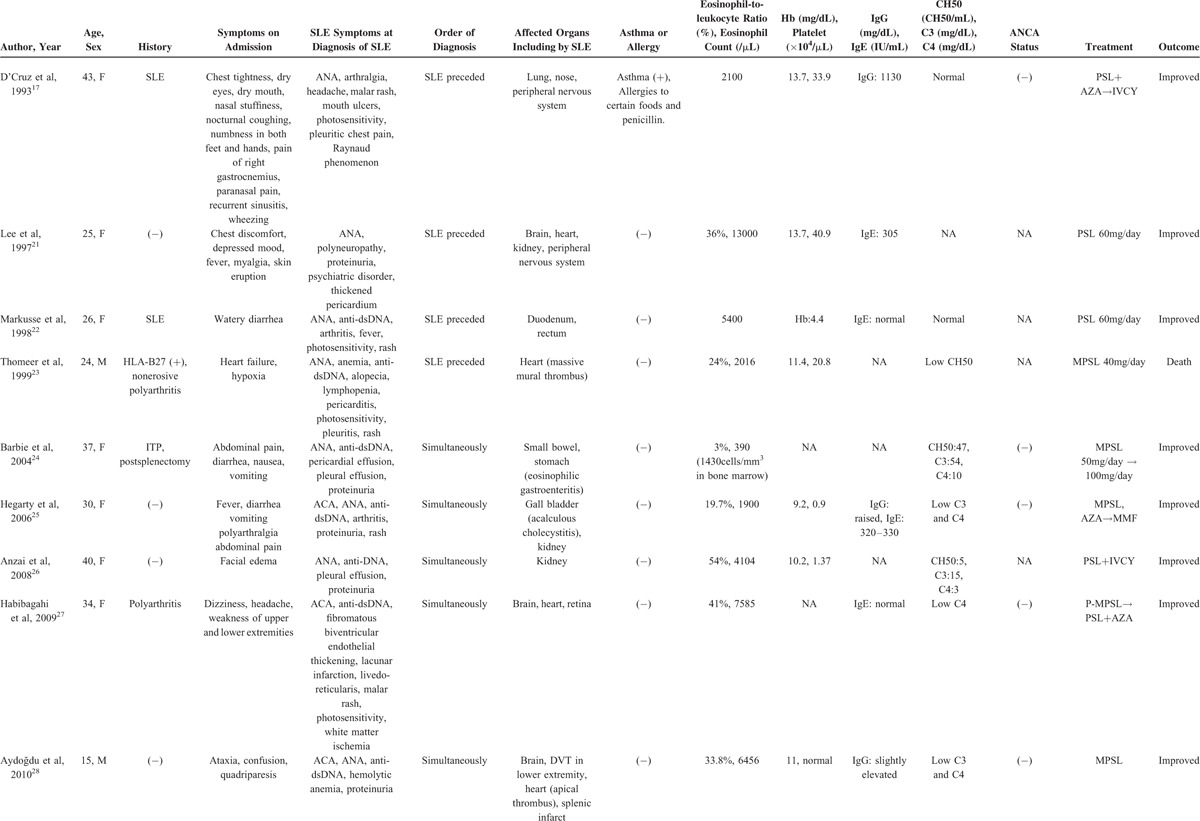
SLE With Eosinophilic Granulomatosis With Polyangiitis or Hypereosinophilic Syndrome

**TABLE 1 (Continued):**
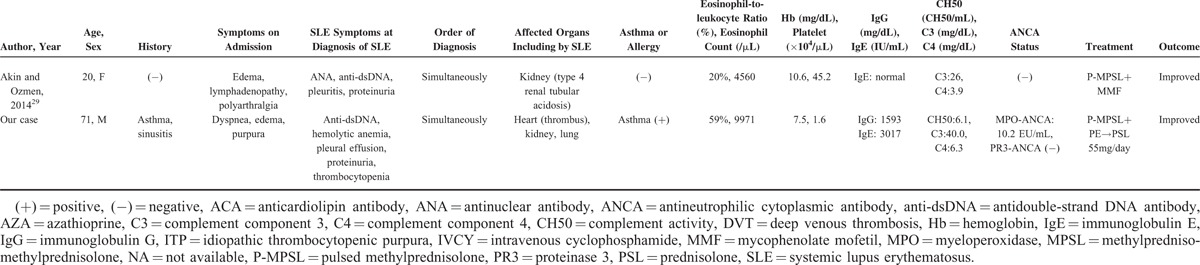
SLE With Eosinophilic Granulomatosis With Polyangiitis or Hypereosinophilic Syndrome

Regarding heart complications, 5 patients had a heart complication including intracardial thrombus. This is more frequent compared with those in patients with HES, because only 5% of HES patients are reported to have cardiac symptoms.^18^ Considering the frequency of heart complications with SLE (pericarditis: 12%–48%, myocarditis: 10%–40%, congestive heart failure: 7%–36%^19^), the heart complications that are seen in EGPA or HES patients with SLE may be affected by elements of SLE rather than those of EGPA or HES. In our patient, the TMA, thrombocytopenia, and heart complication may reflect elements of SLE, and not only EGPA.

On the contrary, 6 of the 11 reported patients had pleural effusion. In SLE, pleural effusion is seen in approximately 24% of patients, not only at diagnosis but also at any time.^20^ SLE with EGPA or HES may have more pleural effusion than simple SLE. Clinical manifestations of eosinophilia with SLE or SLE with eosinophilia may differ from simple SLE or simple eosinophilia.

In conclusion, we have described a patient with EGPA whose case was complicated with TMA. He also fulfilled the SLE criteria, and elements of SLE may have affected his clinical manifestations. Further studies regarding the clinical manifestations and prognosis of EGPA, TMA, and SLE are needed.^21,22,23–29^

## CONSENT

Written informed consent was obtained from the patient for the publication of this case report. A copy of the written consent is available for review by the Editor of this journal. This case report was approved by the Investigation and Ethics Committee at Nagasaki University Hospital.
